# An Automated Microscopic Malaria Parasite Detection System Using Digital Image Analysis

**DOI:** 10.3390/diagnostics11030527

**Published:** 2021-03-16

**Authors:** Jung Yoon, Woong Sik Jang, Jeonghun Nam, Do-CiC Mihn, Chae Seung Lim

**Affiliations:** 1 Department of Laboratory Medicine, Korea University College of Medicine, Seoul 08308, Korea; unoaotro@korea.ac.kr (J.Y.); plasmid18@hanmail.net (W.S.J.); 2 Department of Song-Do Bio-Environmental Engineering, Incheon Jaeneung University, Incheon 21987, Korea; jhnam77@gmail.com; 3 Department of Diagnostic Immunology, Seegene Medical Foundation, Seoul 04805, Korea; dsmin@mf.seegene.com

**Keywords:** malaria, microscopy, parasitemia, automation, *P. falciparum*, *P. vivax*

## Abstract

Rapid diagnosis and parasitemia measurement is crucial for management of malaria. Microscopic examination of peripheral blood (PB) smears is the gold standard for malaria detection. However, this method is labor-intensive. Here, we aimed to develop a completely automated microscopic system for malaria detection and parasitemia measurement. The automated system comprises a microscope, plastic chip, fluorescent dye, and an image analysis program. Analytical performance was evaluated regarding linearity, precision, and limit of detection and was compared with that of conventional microscopic PB smear examination and flow cytometry. The automated microscopic malaria parasite detection system showed a high degree of linearity for *Plasmodium falciparum* culture (R^2^ = 0.958, *p* = 0.005) and *Plasmodium vivax* infected samples (R^2^ = 0.931, *p* = 0.008). Precision was defined as the %CV of the assay results at each level of parasitemia and the %CV value for our system was lower than that for microscopic examination for all densities of parasitemia. The limit of detection analysis showed 95% probability for parasite detection was 0.00066112%, and a high correlation was observed among all three methods. The sensitivity and specificity of the system was both 100% (*n* = 21/21) and 100% (*n* = 50/50), respectively, and the system correctly identified all *P. vivax* and *P. falciparum* samples. The automated microscopic malaria parasite detection system offers several advantages over conventional microscopy for rapid diagnosis and parasite density monitoring of malaria.

## 1. Introduction

Malaria is one of the most dangerous infectious diseases and causes 229 million infections and 409,000 deaths worldwide, according to the World Health Organization (WHO) estimates [1]. Early and accurate diagnosis of malaria is essential for effective treatment and to reduce morbidity and mortality. Therefore, the WHO recommends malaria diagnostic tests for all patients suspected of malaria infection before treatment administration, thereby prompting an increase in the demand of tests to 1 billion by 2020 [2].

Microscopic examination of Giemsa-stained peripheral blood (PB) smears is considered the gold standard for malaria diagnosis. Implementation of other diagnostic methods, including rapid diagnostic tests (RDTs) [3,4] and polymerase chain reaction (PCR) [5,6], has increased for malaria diagnosis, but microscopic confirmation is warranted to obtain additional information such as species identification and parasitemia determination.

Parasite density serves as one of the diagnostic criteria for severe malaria infection and its monitoring is important for the diagnosis and treatment of malaria. Parasite density is associated with patient prognosis [7,8,9]. Parasitemia is one of the essential indicators of the therapeutic effects of antimalarial drugs, and daily blood smear analysis is recommended to document the decrease in parasite density until the absence of parasite on treatment day 7 [7]. With emergence of resistance to available antimalarial drugs, accurate quantitation of parasitemia is required for malaria parasite clearance determination following anti-malarial treatment [10,11]. Microscopic examination of thick and thin blood smears is the standard method for parasitemia measurement. In thick-smear analysis, parasites and 200 or 500 white blood cells (WBCs) are counted according to the number of parasites found, whereas thin blood smears are evaluated for the percentage of infected red blood cells (RBCs) after counting a minimum of 500 to 2000 RBCs [12].

Parasitemia determination by microscopic examination has some limitations in that the method is labor-intensive, requires expert staff, and is highly dependent on the performance of the observer, leading to variability and inconsistency in results [13,14,15]. The quality of Giemsa-stained PB smear may be variable and is another factor in inconsistent parasite density monitoring [12,16].

To overcome these problems, researchers have explored automated malaria detection and parasitemia assessment techniques based on the recognition of digital images using artificial intelligence algorithms to enhance the efficacy of the microscopic examination of malaria parasites. Previous studies have mostly focused on the development of a computer vision algorithm to analyze the microscopic images of Giemsa-stained thin or thick blood smears for malaria diagnosis [17,18,19,20,21,22,23]. Considering that malaria is prevalent in remote areas, the ideal automated microscopic technique for malaria detection should be fully automated with simple sample preparation steps, ease of processing, and simple interpretation of results. Only few studies have worked toward developing a complete system for malaria detection and quantitation consisting of automated digital microscope, sample preparation steps for malaria detection, and image analysis algorithms and these systems have been reported to demonstrate a broad range of sensitivities from 75% to 100% and specificities from 70% to 100% [24,25,26,27].

Here, we describe an automated microscopic detection system for malaria detection and parasitemia determination. The system includes the following four elements: a fluorescent dye for malarial parasite staining, a plastic chip, an automated microscopy platform, and an image analysis algorithm for malaria detection and parasitemia determination. Although the standard method for malaria diagnosis is Giemsa staining, fluorescent staining for malaria is very fast (about 15 min), and has yielded better sensitivity and accuracy owing to the higher contrast of the malarial parasite against the background and lower stain variability. After optimization of test conditions, the analytical performance of this new system was evaluated using *Plasmodium falciparum* culture and malarial parasite-infected samples and was compared to that of conventional microscopy and flow cytometric parasitemia determination.

## 2. Materials and Methods

### 2.1. Materials

#### 2.1.1. Patient Samples

A total of 21 malaria positive samples (3 *P. falciparum* and 18 *Plasmodium vivax*) and 50 control samples were collected at the Korea University Guro Hospital from June 2017 to July 2020. We included samples of patients who were suspected of malaria but tested negative as control samples. Both malaria positive and negative samples included in the study were diagnosed by conventional microscopy and confirmed by PCR. All samples were collected as ethylenediaminetetraacetic acid (EDTA) anticoagulated venous blood specimens and were processed immediately. Parasitemia of *P. falciparum* positive patient samples ranged from 0.05% to 4.75% and the parasitemia of *P. vivax* positive patient samples ranged from 0.03% to 0.22%. The study was conducted in accordance with the guidelines of the Declaration of Helsinki, and was approved by the Institutional Review Board of Korea University Guro Hospital (2017GR0769, 2020GR0329).

#### 2.1.2. *P. falciparum* Culture

*P. falciparum* strain 3D7 was cultured in human O+ RBCs at 3% hematocrit in a medium containing RPMI, 25 mM HEPES buffer, 1.36 g/L hypoxanthine, 0.016 mM thymidine, 7.5% sodium bicarbonate, 20% glucose, 1 M sodium hydroxide, 20% Albumax, and 40 μg/mL gentamicin. The cultures were incubated at 37 °C in the presence of 5% CO_2_, 5% O_2_, and 90% N_2_.

Dilution of malaria culture to various parasite concentrations was carried out as follows: Malaria culture was centrifuged for 2 min and the precipitate was suspended twice in phosphate-buffered saline (PBS). The whole blood sample obtained from a healthy volunteer without malaria infection was also diluted to a hematocrit level of 3%. The resuspended malaria culture and diluted whole blood sample were mixed and the adjusted to various parasite concentration (1.56%, 3.29%, 4.92%, 7.27%, and 8.03%). Dilution of malaria culture to various parasite concentrations was immediately carried out and parasite concentrations in each diluted sample were measured by microscopic examination of Giesma-stained thick and thin PB smears immediately before and after dilutions. To assess the lower limit of detection (LOD), lower parasitemia samples were prepared by serial dilution (0.005%, 0.0025%, 0.0018%, 0.0015%, 0.00125%, 0.0010%, 0.000625%, 0.00050%, 0.000313% and 0.00009%), and the parasite concentrations were calculated accordingly.

### 2.2. Methods

#### 2.2.1. Microscopic Examination of Malaria

The conventional microscopic examination for malaria diagnosis was performed by the Giemsa-stained thick and thin PB smears under 1000× magnification. Thick and thin PB smears were prepared on glass microscopic slides using venous blood collected into K_2_EDTA tubes (Becton Dickinson, Sunnyvale, CA, USA). Parasitemia was determined by counting parasites per 200 or 500 WBCs, depending on the number of parasites. Parasitemia was calculated on the basis of the patient’s WBC count. The number of parasites per μL was divided by the patient’s RBC count to calculate the % infected RBCs. In a thin PB smear, the percentage of infected RBCs was estimated after counting a minimum of 20 fields of the thin film [12,28].

#### 2.2.2. Flow Cytometric Enumeration of Malaria

Parasitemia was estimated with flow cytometry using SYBR Green I and CD235a-PE. In brief, the malaria-infected RBCs were washed with PBS, and 50 μL of the washed sample was incubated with 25 μL of 1:1000 diluted SYBR Green I and CD235a-PE for 15 min at room temperature. Before analysis, the stained samples were washed twice with PBS and resuspended in 1 mL of PBS. For analysis, the RBCs were gated by the forward light scatter (FSC) versus side scatter (90°) (SSC) dot plots, and over 5000 events were acquired for each dot plot. The green and red fluorescence (FL1, FL2) was used to determine malaria-infected RBCs. Parasitemia was estimated by analyzing scattergrams from the computer software and by calculating the infected RBC percentage among the gated RBCs from FL2.

#### 2.2.3. Automated Microscopic Malaria Parasite Detection System

**Figure 1:**
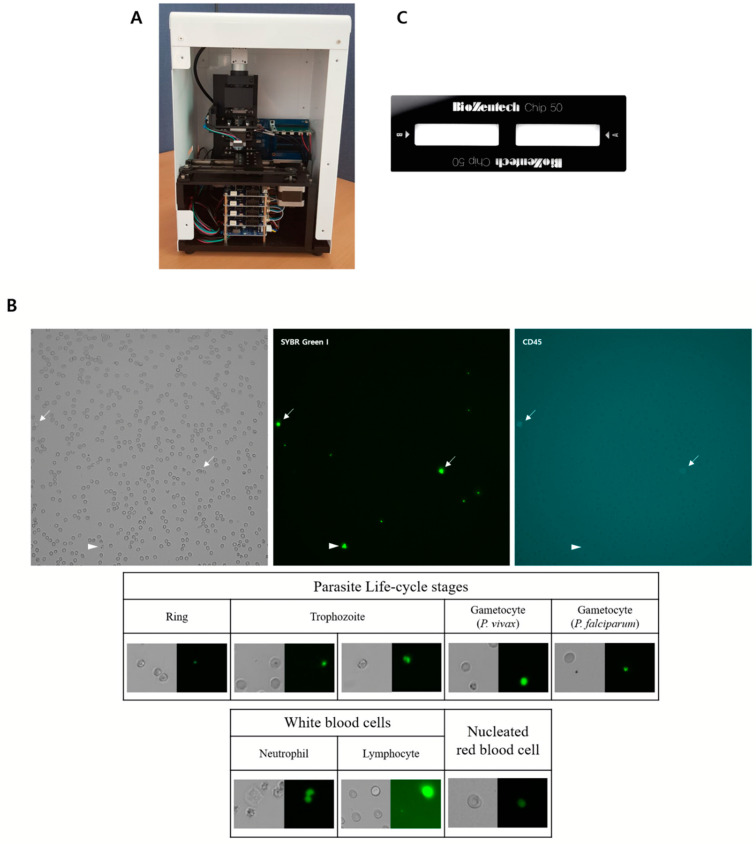
The automated microscopic malaria parasite detection system. (**A**) Automated microscopic malaria parasite detection system hardware. (**B**) Image of stained malarial parasite-infected blood cells showing malarial parasites, white blood cells (WBCs) and nucleated red blood cells obtained using the automated microscopic malaria parasite detection system at 40× magnification (1200 × 1200 pixel^2^). The image is obtained at green LED (upper left), blue LED (upper middle) and red LED (upper right) are shown. The arrows indicate WBCs and the arrow heads indicate gametocyte. The example images of *P. vivax* at different stages, *P. falciparum* gametocyte and WBCs are also shown. (**C**) The plastic chip for the automated microscopic malaria parasite detection system.

The automatic microscopic malaria parasite detection system is an assay platform comprising a digital microscope with an automated scanning and image analysis software. The main body of the automated microscopic malaria parasite detection system is 168 (W) × 252 (D) × 360 (H) mm in size and weighs 8 kg (Figure 1A). The optical system of the automated microscopic malaria parasite detection system uses three different light-emitting diodes (LEDs) as light sources, including green (510–550 nm), blue (455–485 nm), and red (590–650 nm) LEDs. The green, blue and red LEDs are used for RBC, malarial parasite and lymphocyte identification, respectively. The light signal is collected by a 10× or 40× objective lens and passed through a filter block to a charge-coupled device (CCD) camera to obtain images with dimensions of 1200 × 1200 pixel^2^ (Figure 1B). The CCD camera is capable of automated focusing and can scan the plastic chip for the determined length or directly navigate to the determined position. For each sample, 20 frames of images are recorded and the total scan time per sample is 5 to 7 min. In case of low-parasitemia samples (less than five infected RBCs observed for each assay), the automated microscopic malaria parasite detection system continues to count a total of 80 frames. When 80 frames are examined, overall 18,000 to 25,000 RBCs are imaged and analyzed under 40× objective lens and 72,000 to 100,000 RBCs under 10× lens.

#### 2.2.4. Plastic Chip

The plastic chip (Biozentech, Seoul, Korea) is a disposable chip made of a plastic optical material, polymethylmethacrylate (PMMA). The chip is 75 (W) × 25 (D) mm in size and 1.65 mm in height and contains two microfluidic channels of dimensions 27 (W) × 7.4 (D) × 0.05 (H) mm (Figure 1C). Each channel corresponds to 25 μL of sample volume, and the loading time for each channel is 3 to 5 s.

#### 2.2.5. Staining Methods for Automated Microscopic Malaria Parasite Detection System

The malaria parasites were initially stained using SYBR Green I (Invitrogen, Eugene, OR, USA), a fluorescent dye that stains nucleic acid and other nucleic acid-containing structures such as leukocyte nuclei were also stained as well as the malaria parasites. Some small lymphocytes and gametocytes of *P. vivax* may be similar in size when stained using SYBR Green I. For the differentiation of small lymphocytes and gametocytes of *P. vivax*, we applied APC-conjugated monoclonal antibodies against the CD45 antigen (Becton Dickinison, San Jose, CA, USA). The type and concentration of the fluorescent dye was determined through an optimization process. In total, 10 μL of the blood samples was incubated for 15 min at room temperature with 10 μL of 1:1000 diluted SYBR Green I and 5 μL of three fluorochrome-conjugated monoclonal antibodies against the CD45 antigen, and 25 μL of the sample was dispensed into the plastic chip for analysis.

#### 2.2.6. Image Analysis System for Malaria Detection and Parasitemia Determination

Automated system for malaria detection and parasitemia determination was developed and validated using microcopist confirmed image set of *P. falciparum* and *P. vivax.* For malaria diagnosis and parasitemia calculation, the light source was switched between green, blue and red in one field of view and three images were obtained for each light source. RBCs and malarial parasites were detected and counted from the images obtained for the green and blue light sources, respectively.

**Figure 2:**
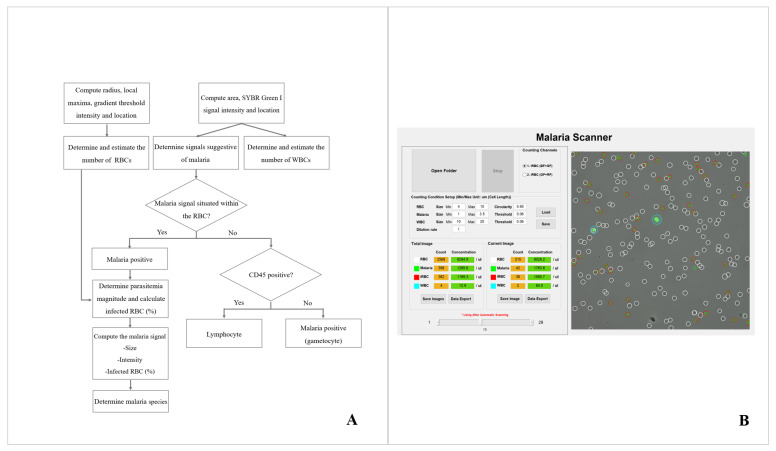
The image analysis system for malaria detection and parasitemia determination. (**A**) The algorithm for malaria detection and parasitemia determination, and (**B**) image analysis result for automatically calculated parasite concentration. Along with the calculated parasite concentration, the analyzed images are shown. Green and white circle represents the identified malaria and RBCs, respectively. If the malaria signal is located within the RBC, the algorithm identifies as malaria parasites and RBCs, which is indicated by red circle.

RBC detection and segmentation algorithm was developed based on the Circle Hough transform algorithm and RBCs were classified according to the detected radius, local maxima, and gradient threshold. For malarial parasite detection, the median filter and adaptive histogram equalization techniques were applied for noise removal and enhancement of image resolution, respectively. The algorithm segmented the parasite signal using connected-component labeling, and classified as the malaria signal depending on the detected area. If a signal suspected to be for malarial parasite matched the coordinates of an RBC in the image obtained from the green light source, it was considered as an infected RBC and the number of these signals was divided by the number of RBCs for parasitemia determination. If the signal suspected to be for malarial parasite was not located within the RBC but CD45 negative, then the signal was considered as malaria parasite, most likely gametocyte (Figure 2A). The image analysis system for the automated microscopic malaria parasite detection system automatically calculates parasite concentration, and the user can verify the results by reviewing the analyzed image (Figure 2B).

#### 2.2.7. Image Analysis System for Malaria Species Classification

**Figure 3:**
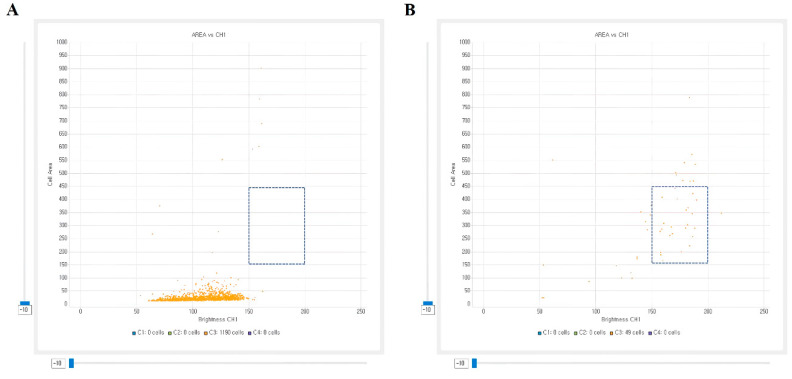
Scatter plots illustrating parasite signal area and SYBR green I intensity level of *P. falciparum* positive sample (**A**) and *P. vivax*-infected patient sample by automatic malaria parasite detection system (**B**). The Cell Area refers to area in pixels and Brightness CH1 refers to signal intensity of SYBR green I. *P. vivax* shows more different parasite developmental stages, such as trophozoites, with lager area and higher intensity compared to that of *P. falciparum.*

*P. vivax* is known to have larger infected RBCs, more frequently observed with Ameboid trophozoites, and exhibits a larger chromatin of parasites than *P. falciparum* [10,11]. However, as the amount of nucleic acids stained with DNA or RNA fluorescent dyes has never been measured previously, we analyzed the signal intensity and measured area of infected RBCs stained with SYBR green I and developed an interspecific classification model to classify the two species. The area of the SYBR green I signal greater than 150 pixels and less than 450 pixels and the intensity level between 150 to 200 was suspected to be at trophozoite stage (Figure 3). The number and the percentage of trophozoite suspected signals was used for species classification between *P. falciparum* and *P. vivax* samples.

#### 2.2.8. Statistics

Linearity was evaluated using simple linear regression between the observed value for parasitemia measured by the automated microscopic malaria parasite detection system and the expected value for parasitemia as measured by microscopic examination of PB smears. Precision was assessed as the percentage coefficient of variation (%CV) at each parasitemia level obtained from repeated measurement, where the %CV was calculated as the ratio of the standard deviation to the mean multiplied by 100. Probit analysis was performed for LOD evaluation using low-parasitemia samples (ranging 0.00009% to 0.005%). The parasitemia measurements by all three methods were compared and analyzed using Pearson’s correlation tests. A value of *p* < 0.05 was considered significant.

## 3. Results

The validation of automated microscopic malaria parasite detection system was achieved using a new set of images obtained from patient samples and malaria culture as described above, independent of the images used for image analysis algorithm validation. A total of 71 samples, including 21 malaria-positive (12 samples at diagnosis and 9 follow-up samples) and 50 malaria-negative samples were collected. When the results obtained by conventional microscopy were considered as true, the automated microscopic malaria parasite detection system showed 100% sensitivity (*n* = 21/21) and 100% specificity (*n* = 50/50). The automated microscopic malaria parasite detection system correctly identified *P. falciparum* (3/3) and *P. vivax* (18/18) using the proposed classification algorithm.

### 3.1. Linearity

**Figure 4:**
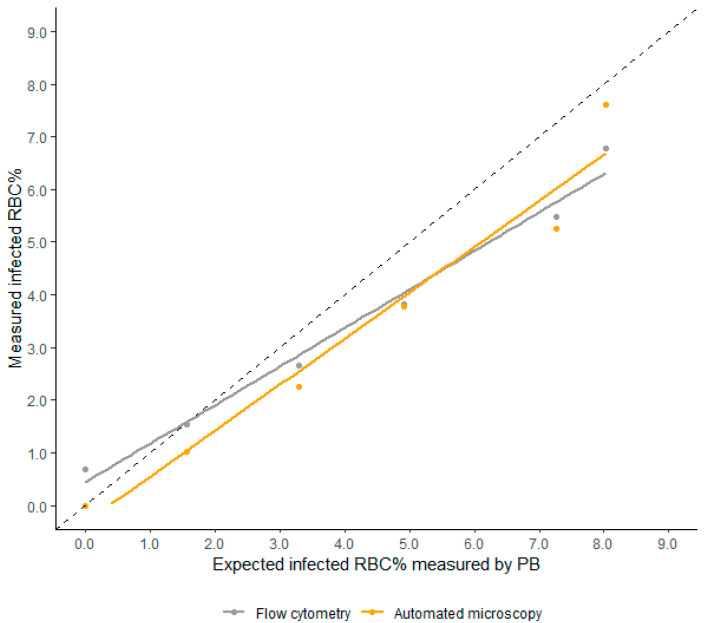
Linearity of percentage infected RBCs evaluated using *P. falciparum* culture. Both the automated microscopic malaria parasite detection system (orange colored) and flow cytometry (gray colored) showed high degree of linearity, with a coefficient of determination (R^2^) of 0.958 (*p* = 0.005) and 0.984 (*p* = 0.001), respectively. The dash-lines indicate a 1:1 line.

The linearity of the percentage of infected RBCs measured by the automated microscopic malaria parasite detection system was evaluated using samples of *P. falciparum* culture with different densities (percentage infected RBCs ranging from 1.56% to 8.03%) and *P. vivax*-infected patient samples (percentage infected RBCs ranging from 0.03% to 0.22%). The linearity of the flow cytometric method was also evaluated using *P. falciparum* culture. For *P. falciparum* culture, the automated microscopic malaria parasite detection system showed a high degree of linearity compared to microscopy, with a coefficient of determination (R^2^) of 0.958 (*p* = 0.005). Flow cytometry also revealed high linearity (R^2^ = 0.984, *p* = 0.001). The automated microscopic malaria parasite detection system showed similar results for *P. vivax* infected patient samples (R^2^ = 0.931, *p* = 0.008) (Figure 4).

### 3.2. Precision

Precision was defined as the %CV of the assay results at each level of parasitemia. Each method used 10 aliquots from one sample with an expected parasitemia level ranging from 1.56% to 8.03% for *P. falciparum* culture and from 0.03% to 0.22% for *P. vivax* infected patient samples. Evaluation of precision of *P. falciparum* detection by the automated microscopic malaria parasite detection system revealed a %CV value ranging from 7.33% to 34.67%. Microscopic examination of PB smears and flow cytometry showed %CV values ranging from 9.41% to 37.07% and 6.98% to 19.29%, respectively. Among the three methods, flow cytometry was the most precise technique for all levels of parasitemia. The automated microscopic malaria parasite detection system was more precise than microscopic examination of PB smear for all levels of percentage infected RBCs (Table 1). Precision evaluation performed using *P. vivax* infected patient samples revealed a %CV value ranging from 28.11% to 53.22% for the automated microscopic malaria parasite detection system and from 33.47% to 55.43% for microscopic examination of PB smears. The %CV value for the automated microscopic malaria parasite detection system was lower than that for microscopic examination for all densities of parasitemia evaluated using *P. vivax* infected samples (Table 2).

### 3.3. LOD Analysis

A total of 30 measurements of the diluted whole blood samples from healthy volunteers, as blank samples, were made to assess limit of blank (LOB). In all blank samples, the percentage infected RBCs was 0%, resulting in an LOB value of 0%. Probit analysis was used for LOD assessment. The LOD for the automated microscopic malaria parasite detection system was estimated by measuring 10 aliquots for each density of parasitemia using *P. falciparum* cultures (percentage infected RBC ranging from 0.00009% to 0.005%). At least one infected RBC counted per sample was considered as a positive result. The automated microscopic malaria parasite detection system showed a positive rate of 100% above 0.0010% but 90% at a concentration of 0.000625%, 80% at a concentration of 0.00050% and 30% at a concentration of 0.0003125%. Probit analysis indicated that 95% probability LOD for parasite detection was 0.00066112% (95% CI, 0.00053780–0.0011873%).

### 3.4. Comparison of Malaria Counting Methods

We compared the results of percentage infected RBCs determined by different malarial parasite counting methods using *P. falciparum* culture and found a high correlation between the automated microscopic malaria parasite detection system and microscopic examination (r = 0.979, *p* = 0.001), the automated microscopic malaria parasite detection system and flow cytometry (r = 0.992, *p* < 0.001), and microscopic examination and flow cytometry (r = 0.996, *p* < 0.001). Comparison between the automated microscopic malaria parasite detection system and microscopic examination using *P. vivax*-infected patient samples also showed a good correlation, but the value was lower than that assessed using the *P. falciparum* culture (r = 0.915, *p* = 0.001).

## 4. Discussion

In the present study, we developed an automated microscopic malaria parasite detection and quantitation system. We propose this system for measuring malarial parasitemia and density monitoring in the clinical setting. After development and optimization of the system, we evaluated its performance in terms of linearity, precision, and LOD.

The sample preparation process for automated microscopic malaria parasite detection system is very fast, and the instrument is easy to use. The estimated manufacturing cost of instrument is about USD 18,000 and the running cost is less than USD 3 per test. The automated microscopic malaria parasite detection system is a platform built using a fluorescent dye for malaria parasite staining. The standard method for malaria diagnosis is Giemsa staining but fluorescent staining for malaria has yielded better sensitivity and accuracy [29]. Moreover, sample preparation using fluorescent dyes is easier and faster (10 min) than Giemsa staining, which takes about 30 min. However, the storage condition for the fluorescent dyes are more complex when compared to that of Giemsa, since the fluorescent dyes are recommended to be stored protected from light and stored at 2–8 °C. We evaluated three fluorescent dyes for DNA and RNA, including SYBR Green I, SYTOX Green, and propidium iodide (PI) as per previous results [29], and selected SYBR Green I owing to its superior fluorescence intensity over those of other dyes (data not shown). Along with the use of a fluorescent dye, the microfluidic chip that automatically disperses the sample in a relatively short time (<5 s) contributes to the simplicity of the sample preparation process.

The performance of the automated microscopic malaria parasite detection system was compared with that of microscopic examination as well as flow cytometry. The results of the automated microscopic malaria parasite detection system showed a good fit to the expected values with a high degree of linearity (R^2^ value of 0.931 and 0.958). The %CV value varied depending on the parasitemia level, but the automated microscopic malaria parasite detection system showed better performance in terms of precision for both *P. falciparum* and *P. vivax* samples. The difference in %CV values was noted, especially in samples with low parasitemia. In case of samples with low parasitemia, microscopic examination could take 20 to 30 min for monitoring parasite density, and it is likely that the automated technology will yield more consistent results, thereby overcoming the variability of results obtained by microscopic examination of PB smears. The performance of flow cytometry was better than that of the automated microscopic malaria parasite detection system for all evaluated aspects, but application of flow cytometry for parasitemia estimation in the clinical setting has many limitations such as difficulty in accessibility and high cost of the instrument as well as each test run.

The determined LOD for the automated microscopic malaria parasite detection system was 0.00066112%. Considering that the normal RBC count range is 4,500,000/μL to 5,000,000/μL, the LOD value can be estimated to be about 30 parasites/μL to 33 parasites/μL. However, increasing the number of scanning images in the automated microscopic malaria parasite detection system over the standard scan (*n* = 80) may further improve its sensitivity. This LOD seems better than those of the previously developed computer image-based automated malaria diagnostic systems and malaria RDTs. The LODs of the World Health Technology autoanalyzer and the Global Good Fund prototype have been estimated to be 140 and 100 parasites/μL, respectively [25,27]. Both these platforms use the standard Giemsa stain for malaria detection, and the lower LOD value observed for the automated microscopic malaria parasite detection system may be attributed to the use of fluorescent dyes. The other two platforms applying fluorescent staining for malaria detection have potentially yielded LOD values of 10 and 50 parasites/μL [24,26], respectively, but these LODs were assumed based on the number of RBCs counted or because the evaluation method was not mentioned. Regarding microscopic examination of malaria, the LOD of the expert microscopist can be as low as 5 parasites/μL, but the average microscopist can detect as low as 50 to 100 parasites/μL [30]. The LOD value of RDT range from 100 to 500 parasites/μL [15,31]. PCR assays were reported to have an LOD value ranging from 1 to 5 parasites/μL [32]. The detection limit of the automated microscopic malaria parasite detection system was higher than the ideal detection limit of the expert microscopist (~5 parasites/μL) but was lower than that of the average microscopist (50 to 100 parasites/μL). The detection limits of microscopic examination of malaria is greatly dependent on the proficiency of the microscopist [30] and to achieve the ideal LOD, the expert microscopist and significant amount of time for blood smear examination is required. Moreover, since we only evaluated the LOD using malaria culture diluted with whole blood, further assessment of LOD using the low parasitemia patient field samples would be required for comparing LOD between automated microscopic malaria parasite detection system and other methods. 

The limitation of our study is that we validated the performance using small number of patient samples along with cultured *P. falciparum* samples. Additional evaluation of sensitivity and specificity is needed using a large number of samples of malaria patients, samples of various malaria species and samples with diverse parasitemia, especially very low parasitemia samples. Assembly of the targeted and extensive data would allow construction of a computer vision platform that could detect all stages and species of malarial parasites, successfully discriminate other false-positive signals, and be applied for malaria screening. Further development of our prototype should focus on discrimination among the different stages of malarial parasites and various parasite species. Moreover, differentiation between possible false-positive signals, blood inclusions such as Howell–Jolly bodies, and nucleated RBCs and other bloodstream pathogens such as *Babesia* species, microfilariae, and trypanosomes should be incorporated into the image analysis algorithm.

## 5. Conclusions

Automated microscopic malaria parasite detection system demonstrated a high degree of linearity and precision in the measurement of density of malaria parasites. The LOD was estimated to be 0.00066112% (30 parasites/μL to 33 parasites/μL), suggesting that automated microscopic malaria parasite detection system may improve the sensitivity of malaria detection as compared with the conventional microscopic method. Taken together, automated microscopic malaria parasite detection system is a rapid, easy-to-use, and accurate method for the detection and monitoring of malaria infection and may serve as a valuable tool in clinical practice and research studies.

## Figures and Tables

**Table 1:** Precision of parasitemia measured with PB smears, the automated microscopic malaria parasite detection system, and flow cytometry using *P. falciparum* malaria cultures for various levels of parasitemia.

Infected RBC (%)	PB Smear		Automated Microscopic Malaria Parasite Detection System		Flow Cytometry	
	Mean ± SD	%CV	Mean ± SD	%CV	Mean ± SD	%CV
8.03	8.03 ± 0.76	9.41	7.61 ± 0.56	7.33	6.78 ± 0.47	6.98
7.27	7.27 ± 0.82	11.34	5.25 ± 0.51	9.63	5.49 ± 0.50	9.03
4.92	4.92 ± 1.18	23.96	3.78 ± 0.54	14.32	3.83 ± 0.39	10.13
3.29	3.29 ± 0.71	21.67	2.25 ± 0.36	16.04	2.66 ± 0.35	13.20
1.56	1.56 ± 0.58	37.07	1.02 ± 0.35	34.67	1.54 ± 0.30	19.29

**Table 2:** Precision of parasitemia measured with PB smears and the automated microscopic malaria parasite detection system using *P. vivax*-positive patient samples for various levels of parasitemia.

Infected RBC (%)	Thick Smear		Thin Smear		Automated Microscopic Malaria Parasite Detection System	
	Mean ± SD	%CV	Mean ± SD	%CV	Mean ± SD	%CV
0.21	0.21 ± 0.08	39.27	0.22 ± 0.22	104.38	0.23 ± 0.09	38.85
0.17	0.17 ± 0.06	33.47	0.13 ± 0.13	99.52	0.16 ± 0.04	28.11
0.06	0.06 ± 0.03	55.43	0.03 ± 0.07	207.87	0.08 ± 0.04	53.22

## Data Availability

Not applicable.
